# Aortic and Mitral Valve Infective Endocarditis Caused by Gemella sanguinis

**DOI:** 10.7759/cureus.28099

**Published:** 2022-08-17

**Authors:** Netra Shah, Diane SInnatamby Moon, Brody Wehman

**Affiliations:** 1 Center for Medical Sciences, Godwin High School, Glen Allen, USA; 2 Infectious Disease, Bon Secours Mercy Health System, Mechanicsville, USA; 3 Surgery, Bon Secours Mercy Health System, Mechanicsville, USA

**Keywords:** mechanical valve, gemella, aortic valve abscess, mitral valve replacement, aortic valve replacement, vv ecmo, gemella sanguinis, infective endocarditis

## Abstract

We present a case of infective endocarditis (IE) of the aortic valve, mitral valve, and aortomitral curtain caused by *Gemella sanguinis. *The patient was an otherwise healthy 53-year-old male without significant risk factors for infective endocarditis in his medical history. Due to the extent of the infective endocarditis and the rapid deterioration of his clinical condition, which included respiratory failure and severe heart failure, the patient was treated with urgent surgery (a Commando operation where both the aortic and mitral valves were replaced and the aortomitral curtain was reconstructed), broad-spectrum antibiotics, and aggressive postoperative measures such as venovenous (VV) extracorporeal membrane oxygenation (ECMO). This is the first reported case where the aortic valve, mitral valve, and aortomitral curtain were affected by *G. sanguinis*.

## Introduction

Infective endocarditis (IE) is uncommon in the general population, with an incidence of roughly 1.7-6.2 cases/100,000 patient-years; however, having a heart condition, such as mitral valve prolapse, degenerative valve disease in the elderly, and previous valvular replacements and interventions, greatly increases the risk [1]. Common bacterial causes of IE include species of Staphylococcus or Streptococcus. A rarer bacterial cause of IE is the Gemella species. We report the first case in the literature of IE caused by *Gemella sanguinis* that affected both the aortic and mitral valves, along with the aortomitral curtain (also known as the intervalvular fibrosa).

As of 2019, there were 12 published cases of IE by the Gemella species. The Gemella genus was discovered in 1960 when it was differentiated from the streptococci group. The Gemella species are facultatively anaerobic, catalase-negative, Gram-positive cocci, non-spore-forming bacteria, and reside in the gastrointestinal tract and the microbiome of the oral cavity [2]. Currently, there are nine species that are recognized by the scientific community; however, seven of those are associated with human infection [2]. These species are *Gemella haemolysans*, *Gemella morbillorum*, *Gemella sanguinis*, *Gemella bergeri*, *Gemella taiwanensis*, *Gemella assacharolytica*, and *Gemella parahaemolysans* [2]. While *G. morbillorum* and *G. haemolysans* are the most frequent causes of IE, *G. sanguinis*, though rare, mainly infects heart valves and the cardiac muscle.

## Case presentation

A 53-year-old male, previously healthy with a past medical history of hypertension, was admitted with complaints of chest pain for three days, which worsened with activity and was associated with palpitations. A physical examination displayed a blood pressure of 96/36, a pulse of 70, and a temperature of 97.7 °F. His respiratory status declined rapidly, and the patient became hypoxic and was intubated while awaiting a bed. He spiked a fever of 104.5 °F and was tachycardic, becoming hemodynamically unstable, requiring escalating doses of vasopressor support. No risk factors for IE were identified at the time by history and physical examination.

**Figure 1:**
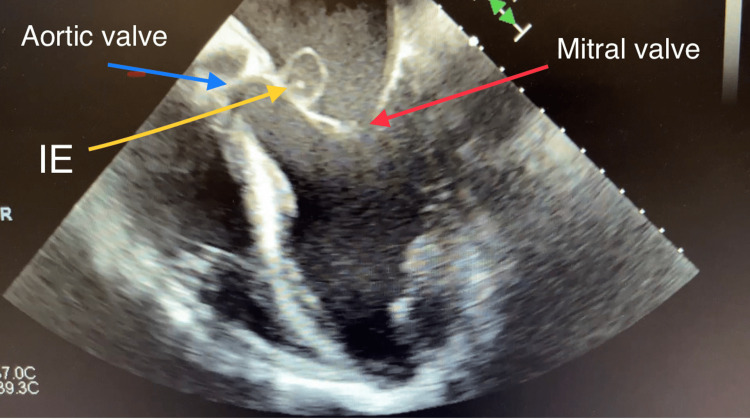
Transesophageal Echocardiogram Showing Infective Endocarditis of Mitral Valve and Aortic Valve The TEE shows extensive endocarditis eroding the aortomitral curtain with a fistula forming between the left ventricle and left atrium.

**Figure 2:**
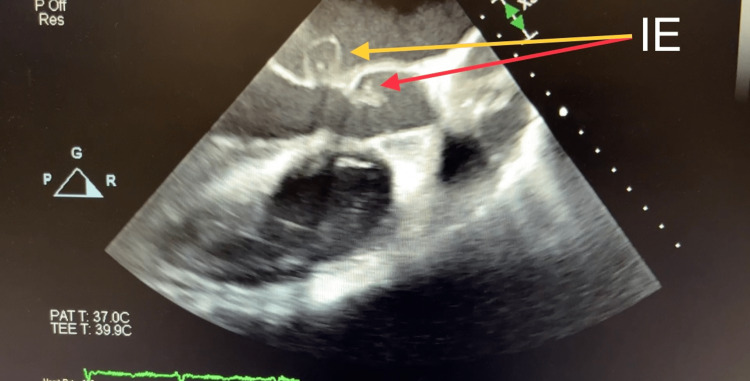
Transesophageal Echocardiogram Showing Infective Endocarditis Involving Aortic Valve and Forming a Fistula in the Aortomitral Curtain

**Figure 3:**
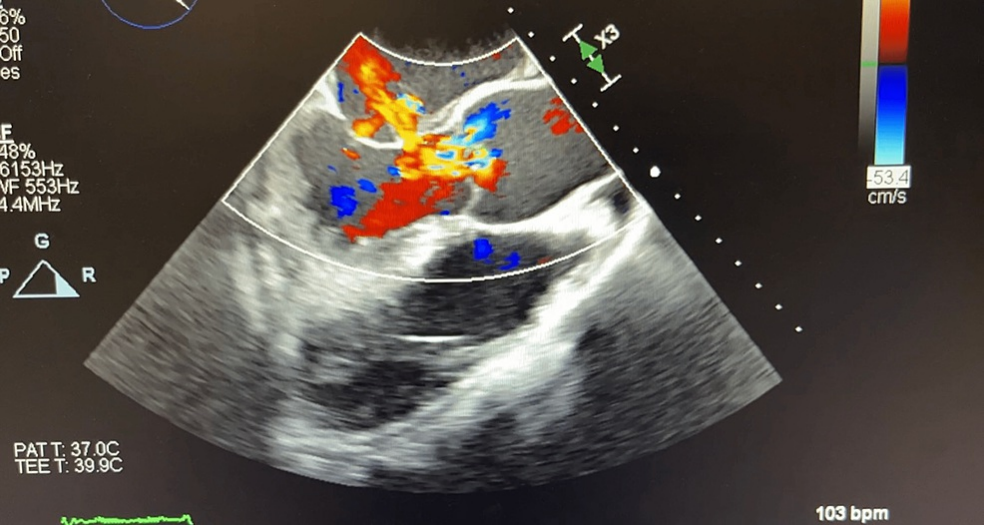
Transesophageal Echocardiogram Showing Severe Aortic Regurgitation, Diastolic Mitral Regurgitation, and Anterior Mitral Leaflet Perforation

**Figure 4:**
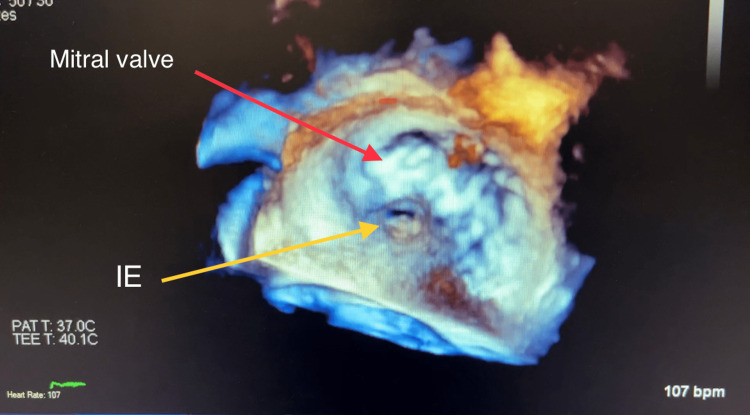
Three-Dimensional Transesophageal Echocardiogram Image Showing Infective Endocarditis of Mitral Valve With Anterior Leaflet Perforation

Computed tomography (CT) of his chest displayed a ground-glass appearance, which suggested pulmonary edema. This was asymmetrically distributed; the right was greater than the left. There was no evidence of pulmonary embolism. The computed tomographic angiography (CTA) suggested a splenic infarct (dimensions: 4.7 cm × 2.4 cm × 4.9 cm) and was confirmed by abdominal ultrasound. Bilateral small pleural effusions were also shown on the CTA. The EKG showed the ventricular rate and atrial rate to be 78 BPM. A stat echocardiogram displayed near normal wall thickness with isolated dyskinesis of the apical inferior segment. The estimated LV ejection fraction was 60-65%. There was severe eccentric paravalvular regurgitation involving the mitral valve and the echo exhibited large vegetation, measuring 1.6 cm × 1.1 cm, on the atrial side of the anterior mitral valve leaflet. The left atrium was mildly dilated, measuring 5 cm × 6 cm, with the annulus of the aorta and ascending aorta being normal-sized. The aortic root was mildly dilated and mild anterior and posterior pericardial effusion were noted on the echo. The tricuspid valve and pulmonic valve structures were both normal. The transesophageal echocardiogram (TEE), done on the same day in the ICU after the patient was intubated, displayed the aortic valve annulus measuring 2.7 cm with no aortic valve stenosis. However, there was severe aortic insufficiency due to probable rupture of the non-coronary cusp with a fistula into the left atrium due to the destruction of the aortomitral curtain. The mitral valve annulus was dilated and the ruptured abscess of the A2 leaflet resulted in severe mitral insufficiency (Figures 1-4 and Videos 1-5).

**Video 1:** Two-Dimensional Transesophageal Echocardiogram Four Chamber View Showing Infective Endocarditis 2D TEE, four chamber view showing infective endocarditis causing destruction of aortomitral curtain and creating a fistula between left ventricle and left atrium.

**Video 2:** Two-Dimensional Transesophageal Echocardiogram of Left Ventricular Outflow Tract View Showing Infective Endocarditis of Aortic Valve and Fistula 2D TEE left ventricular outflow tract long axis view showing infective endocarditis of aortic valve and fistula between left ventricle and left atrium.

**Video 3:** Transesophageal Echocardiogram Showing Severe Aortic Regurgitation and Diastolic Mitral Regurgitation Through Anterior Leaflet Perforation 2D color doppler flow of LVOT long axis view showing severe mitral regurgitation due to anterior leaflet perforation and severe aortic regurgitation caused by infective endocarditis of the aortic valves.

**Video 4:** Transesophageal Echocardiogram Showing Severe Mitral Regurgitation 2D TEE, four chamber view with color flow doppler showing severe mitral valve regurgitation due to anterior leaflet perforation.

**Video 5:** Three-Dimensional Transesophageal Echocardiogram Showing Fistula Due to Infective Endocarditis of Anterior Mitral Valve 3D reconstruction showing infective endocarditis of anterior mitral valve leaflet and fistula between left ventricle and left atrium.

Multiple blood cultures were done over several days. Blood cultures done on initial presentation showed *G. sanguinis* growing from two out of four bottles. The bottles were sent to the reference lab for sensitivities. The nasal PCR test for methicillin-resistant *Staphylococcus aureus* (MRSA) was negative and the respiratory cultures were normal. There was no growth in the urine culture. Blood cultures on the following day were negative.

Due to the patient’s acute respiratory failure and hemodynamic collapse from acute severe aortic regurgitation and mitral regurgitation, the patient was taken urgently to the operating room on multiple pressors. A "double valve-double patch repair" (commando procedure), described in detail elsewhere [3], was performed with an aortic valve replacement with a 27 mm mechanical valve and a mitral valve replacement with a 33 mm mechanical valve. The aortomitral curtain and dome of the left atrium were reconstructed using bovine pericardium. The commando procedure is a very technically challenging procedure where the aortic and mitral valves are replaced, and the intervalvular fibrous body (aortomitral curtain) is reconstructed [3]. Despite an expedient and technically successful operation, the patient had fulminant pulmonary edema and multiorgan failure post-bypass. Post-bypass TEE examinations showed moderate left ventricular dysfunction with an ejection fraction of 30% and moderate right ventricular dysfunction with right ventricular dilation. The replaced aortic valve and mitral valve were functioning normally with normal gradients and no paravalvular leaks. Peripheral venovenous (VV) extracorporeal membrane oxygenation (ECMO) was required to support the patient’s oxygenation and ventilation. The patient was then transferred to the ICU. Postoperative studies showed a progressive increase in lactic acid levels and a decrease in urine output and hemoglobin levels. The patient died four days after the first examination.

Blood and tissue cultures performed during surgery displayed moderate growth in the native aortic valve and light to moderate growth in the aortomitral curtain. Tissue cultures displayed light *G. sanguinis* on the aortic valve and the aortomitral curtain. However, there was moderate growth in the mitral valve. Fungal tissue cultures were negative. The pathology of the aortic valve displayed myxoid change and mild chronic inflammation. Broad antibiotic coverage with vancomycin, cefepime, and doxycycline was continued, and anidulafungin was added pending sensitivities for *G. sanguinis*. The hematology and chemistry findings of the patient are shown in Table 1, and the temperature findings of the patient are shown in Table 2.

**Table 1:** Hematology and Chemistry Labs of Patient Over Various Days

Date		3/2	3/3	3/4	3/5	3/6
Hematology	WBC count in thousands	11.7	15.7	12.9	47	58.5
	Differential count (neutrophil, lymphocytes)	N:81,L:11			N:92,L:4	
Chemistry	BUN	19	25	24	38	30
	Creatinine	1.27	1.26	1.08	3.27	3.25
	ALT	27	43	73	578	>3500
	AST	22	56	78	1295	>2000
	Alk phosphatase	79	64	63	62	148
	Bilirubin total	1.2	1.0	1.1	1.5	4.7
	Procalcitonin	45.93		61.0		

**Table 2:** Temperature of Patient Over Various Days

Date	3/2	3/3	3/4	3/5
Temperature (℉)	104	100.4 (in ICU)	100.4 (t_max_ was 100.4 after 24 hours)	97.5-97.6 (max temp post surgery was 98.7)

## Discussion

The risk factors, predisposing factors, and causes of infective endocarditis have changed tremendously in the past century. A relatively new and rare cause of IE is the Gemella species, which was recently differentiated from the streptococci group. The species is a Gram-positive coccus, facultatively anaerobic, non-spore-forming, and catalase negative. In humans and warm-blooded animals, they are mainly found in the oral cavity. Cases of IE by this genus are extremely rare and, up till 2019, there have been only 12 published cases of IE. Out of those 12 cases, seven of them had a past dental history like dental abscess and dental procedures, six of them had cardiac abnormalities such as previous mechanical valvular replacements, rheumatic heart disease, and repaired septal defect, and one of them had diabetes and was on dialysis [2,4]. About 80% of the cases had infected native valves, and 75% of the patients were males. Reports from the past show that IE is more likely to infect the aortic valve than the mitral valve [2]. Of the 12 past cases, nine of them recovered fully. Most of them were given various antibiotics/antimicrobial treatments or cardiac valve surgeries coupled with prolonged antibiotic treatments [2]. Most cases were treated with penicillin, beta-lactam + aminoglycan for a six to eight-week period due to the high bacterial density. The fact that the cases had high bacterial density and approximately 75% of the cases reported prolonged antibiotic treatments suggests that the strain is very aggressive and potentially antibiotic-resistant [2,5-7].

Generally, the indications for urgent surgery in the setting of infective endocarditis are refractory heart failure symptoms due to severe valvular dysfunction, persistent infections like fungal infections, perivalvular abscesses and pseudoaneurysms, and cases of high embolic risk [8]. In our case, severe heart failure with respiratory decompensation in the setting of acute severe aortic insufficiency and mitral regurgitation was the indication for emergent surgery. Due to extensive endocarditis involving the mitral and aortic valves with severe damage to the aortomitral curtain, a "commando operation" was performed in which both valves were replaced and the aortomitral fibrous body was reconstructed. Though technically challenging, the outcomes of the "commando operation" in itself are very good, as evidenced by the paper cited by David et al. [3]. In the setting of acute cardiogenic and septic shock, however, the expected mortality for any form of emergency cardiac surgery is quite high.

The Gemella strains are identified through various blood cultures separated by time periods. The previously published cases reported determining the strain by using blood culture and determining that the strain is a Gram-positive coccus and is catalase negative. The physicians identified the isolates by the VITEK 2 automated system, and molecular confirmation of the isolates was done by using PCR and sequencing the 1289rt from the 16Sr RNA gene [2].

## Conclusions

Infective endocarditis due to atypical bacteria is a growing problem. Our case demonstrates a relatively healthy patient with no known risk factors developing severe endocarditis due to *G. sanguinis* involving two valves and a paravalvular abscess with subsequent destruction of the aortomitral curtain. Despite being treated aggressively with broad-spectrum antibiotics, early surgery, and heroic postoperative measures including VV ECMO, the patient ultimately did not survive. This illustrates the aggressive nature of *G. sanguinis* and its ability to produce a rapid progression of clinical symptoms and pathology.

## References

[1] Prendergast BD (2006). The changing face of infective endocarditis. Heart.

[2] Maraki S, Plevritaki A, Kofteridis D, Scoulica E, Eskitzis A, Gikas A, Panagiotakis SH (2019). Bicuspid aortic valve endocarditis caused by Gemella sanguinis: case report and literature review. J Infect Public Health.

[3] David TE, Kuo J, Armstrong S (1997). Aortic and mitral valve replacement with reconstruction of the intervalvular fibrous body. J Thorac Cardiovasc Surg.

[4] Emmanouilidou G, Voukelatou P, Vrettos I (2019). A case report of successful conservative treatment for infective endocarditis caused by Gemella sanguinis. Case Rep Infect Dis.

[5] Yang CH, Tsai KT (2014). Gemella sanguinis endocarditis: first case report in Taiwan and review of the literature. J Formos Med Assoc.

[6] Shukla SK, Tak T, Haselby RC, McCauley CS Jr, Reed KD (2002). Second case of infective endocarditis caused by Gemella sanguinis. WMJ.

[7] Chadha S, Chen O, Shetty V, Sadiq A, Hollander G, Frankel R, Shani J (2013). "Kissing" vegetation in a rare case of infective endocarditis by Gemella sanguinis. Am J Med Sci.

[8] Soud M, Moussa Pacha H, Alraies MC (2018). How soon should patients with infective endocarditis be referred for valve surgery?. Cleve Clin J Med.

